# Pro-adrenomedullin, pro-endothelin-1, procalcitonin, C-reactive protein and mortality risk in critically ill children: a prospective study

**DOI:** 10.1186/cc13064

**Published:** 2013-10-16

**Authors:** Corsino Rey, Irene García-Hernández, Andrés Concha, Pablo Martínez-Camblor, Marta Botrán, Alberto Medina, Belén Prieto, Jesús López-Herce

**Affiliations:** 1 UCI Pediátrica, Departamento de Pediatría, Hospital Universitario Central de Asturias, Celestino Villamil s/n, Oviedo 33006, Spain; 2 Pediatric Service, Complejo Hospitalario Universitario de A Coruña, A Coruña, Spain; 3 Oficina de Investigación Biosanitaria FICYT, University of Oviedo, Oviedo, Spain; 4 Pediatric Intensive Care Department, Hospital General Universitario Gregorio Marañón, Instituto de Investigación Sanitaria Gregorio Marañón, Madrid, Spain; 5 Biochemical Department, Hospital Universitario Central de Asturias, University of Oviedo, Oviedo, Spain

## Abstract

**Introduction:**

We tested the hypothesis that higher mid-regional pro-adrenomedullin (MR-proADM), carboxy-terminal pro-endothelin-1 (CT-proET-1), procalcitonin (PCT) and C-reactive protein (CRP) plasma concentrations would be associated with increased prediction of mortality risk scores.

**Methods:**

Prospective observational study set in two pediatric intensive care units (PICUs). Two-hundred-thirty-eight patients were included. MR-proADM, CT-proET-1, PCT and CRP levels were compared between children with PRISM III and PIM 2 > p75 (Group A; *n* = 33) and the rest (Group B; *n* = 205).

**Results:**

Median (range) MR-proADM levels were 1.39 nmol/L (0.52–12.67) in group A versus 0.54 (0.15–3.85) in group B (*P* < 0.001). CT-proET-1 levels were 172 pmol/L (27–500) versus 58 (4–447) (*P* < 0.001). PCT levels were 7.77 ng/mL (0.34–552.00) versus 0.28 (0.02–107.00) (*P* < 0.001). CRP levels were 6.23 mg/dL (0.08-28.25) versus 1.30 mg/dL (0.00-42.09) (*P* = 0.210). The area under the ROC curve (AUC) for the differentiation of group A and B was 0.87 (95% CI:0.81–0.821) for MR-proADM, 0.86 (95% CI:0.79–0.92) for CT-proET-1 and 0.84 (95% CI:0.74–0.94) for PCT. A MR-proADM > 0.79 nmol/L had 93% sensitivity and 76% specificity to differentiate groups, whereas a CT-proET-1 > 123 pmol/L had 77% sensitivity and 84% specificity, and a PCT concentration > 2.05 ng/mL had 80% sensitivity and specificity.

**Conclusions:**

In critically ill children, high levels of MR-proADM, CT-proET-1 and PCT were associated with increased prediction of mortality risk scores. MR-proADM, CT-proET-1 and PCT concentrations higher than 0.80 nmol/L, 123 pmol/L and 2 ng/mL, respectively, could be used by clinicians to identify critically ill children at higher prediction of risk death scores.

## Introduction

Having available tools to determine the prognosis of critically ill children at admission to the Pediatric Intensive Care Unit (PICU), or during the first 24 hours after admission, constitutes a clinical necessity. The better evaluated tools have been scales developed to estimate the mortality risk depending on clinical signs and routine analysis. The most used scales are Pediatric Risk of Mortality (PRISM III) and Pediatric Index of Mortality (PIM 2) [1-5].

Procalcitonin (PCT) was initially used to determine sepsis diagnosis [6] and, afterwards, to help in severity classification of patients [7] and to guide antibiotic treatment duration [8]. C-reactive protein (CRP) rises in response to infectious and inflammatory diseases and shows greater elevations in serious bacterial [6,7,9,10] infections. CRP has been shown to be elevated in adult patients with a higher mortality risk [11,12].

Recently, new readily measurable circulating biomarkers have been described as an additional tool for severity classification of septic patients and prediction of mortality in critically ill patients. Adrenomedullin (ADM) and endothelin-1 (ET-1) are included among these new biomarkers [13-21]. ADM is mainly released from endothelial cells, acts as a potent vasodilator and has natriuretic effects [22,23]. Other ADM properties include a reduction in endothelial permeability [24], bactericidal effects and down-regulation of pro-inflammatory cytokines [25]. ET-1 is a potent vasoconstrictor agent, synthesized mainly by endothelial cells. Elevated levels of ET-1 were found during systemic infections [16] and ET-1 levels correlated with mortality risk [21]. Recently, assays have become available to determine circulating mid-regional pro-adrenomedullin (MR-proADM) and carboxy-terminal pro-endothelin-1 (CT-proET-1) concentrations [26,27]. These peptides are co-synthesized with ADM and ET-1, respectively, and have the advantage of a longer half-life, lack of bioactivity and lack of protein binding, which makes them more suitable for daily practice [28]. Briefly, on admission, concentrations of MR-proADM and CT-proET-1 were shown to increase gradually in correlation with severity ranging from systemic inflammatory response syndrome (SIRS) to severe forms of sepsis or with the pneumonia severity index (PSI) [13,14,21].

So far, no data are available for pediatric patients. Therefore, the objective of our study was the investigation of MR-proADM, CT-proET-1, PCT and CRP levels in a well-defined cohort of consecutive PICU patients to test the hypothesis that higher plasma concentrations would be associated with increased prediction of mortality risk scores in critically ill children. As a secondary objective, we also tested the hypothesis that higher plasma marker concentrations would be associated with a number of organ failures >1.

## Materials and methods

We designed a prospective observational study set in two PICUs of University Hospitals (eight-bed PICU of Hospital Universitario Central de Asturias in Oviedo and eleven-bed PICU of Hospital Universitario Gregorio Marañón in Madrid). The study protocol was approved by the Hospital Ethics Committee of Hospital Universitario Central de Asturias. Written informed consent was obtained from the patients’ parents or guardians and from children above 12 years old.

The study was conducted in 254 consecutive patients, age <18 years, who were admitted to one of these PICUs. The exclusion criteria were no blood extraction during the first 12 hours and parents, guardians or children above 12 years old who did not consent to participate. A total of 56 patients were excluded for these reasons. The following variables were prospectively recorded at admission: age, weight, cause of PICU admission, diagnosis and previous diseases. Respiratory rate (RR), heart rate (HR), blood pressure (BP), O_2_ saturation (Sat O_2_), urine rate and administration of vasopressor agents were recorded hourly. Radiographic and microbiologic diagnostics were performed when indicated. Blood cultures were performed when there was clinical suspicion of infection or if a patient’s temperature was >38°. The PIM 2 value was calculated at admission and PRISM III during the first 12 hours after admission, as it was the normal clinical practice. PIM 2 and PRISM III had been previously validated in both PICUs [1]. Biochemical routine determinations including CRP and PCT were performed during the first 12 hours after admission. Venous blood samples were collected in tubes containing ethylenediaminetetraacetic acid (EDTA). A plasma aliquot was frozen and stored at -80°C for further determination of MR-proADM and CT-proET-1.

### Mortality risk groups

Patients were divided into two groups according to mortality risk scores. The higher score risk mortality group (Group A) included patients with a PIM 2 and PRISM III score > p75 (n = 33); the lower score risk mortality group (Group B) included patients with a PIM 2 and/or PRISM score ≤ p75 (n = 221).

### Number of organ failures groups

Patients were divided into two groups according to the number of organ failures (cardiovascular, respiratory, neurological, hematological, renal and hepatic) following conference consensus criteria [29]. Group A1 included patients with a number of organ failures >1 (n = 71) whereas group B1 included patients with <2 organ failures (n = 182).

### Infectious subgroups

Patients were classified as infectious or non-infectious subgroups during the first 24 hours after admission according to the definitions of the European Society of Intensive Care/Society of Critical Care Medicine [30] modified for use in pediatrics [29].

### Measurement of CRP, PCT, MR-pro-adrenomedullin and CT-proET-1

MR-proADM, CT-proET-1 and PCT were measured in EDTA plasma using a sandwich immunoassay (TRACE technology; Brahms, Hennigsdorf, Germany). Analytical detection limits were 0.08 nmol/L for pro-ADM, 0.4 pmol/L for CT-proET-1 and 0.02 ng/mL for PCT. Plasma CRP was measured on a Modular Analytics Cobacs 6000 (Roche diagnostics, Indianapolis, IN, USA) by an immunoturbidimetric technique. Analytical detection limit was 0.07 mg/dL.

### Statistical analysis

Patients’ clinical and biological parameters were described using frequencies, percentages, medians and ranges. The two groups of patients (A versus B; A1 versus B1) were compared using the non-parametric Mann–Whitney *U*-test for continuous variables and the exact *χ*^2^ test for categorical data. Receiver operating characteristic (ROC) curves and the respective areas under the curve (AUC) were calculated; for these parameters 95% confidence intervals were also reported. The AUCs were compared in order to establish the marker with the highest global diagnostic efficiency to calculate mortality risk scores and number of organ failures. The Youden criterion was used to establish threshold (cut-off) values and to build decision rules to differentiate groups A versus B and A1 versus B1. In addition, in order to measure the quality of these decision rules, a leave-one-out method was developed and the obtained sensitivity, specificity, positive predictive (PPV) and negative predictive (NPV) values are reported. Trying to find a combined score of biomarkers model, a multivariate logistic regression analysis which included a stepwise method based on the likelihood ratio has been explored. A *P*-value <0.05 was considered statistically significant.

## Results

### Baseline characteristics

Two hundred fifty four patients were included in the study (150 boys and 104 girls).

Baseline demographic, clinical and laboratory characteristics of the patients are shown in Table 1. More than half were younger than four years old. The main reasons for PICU admission were postoperative, cardiac surgery, and respiratory and infectious disease. A total of five patients (2%) died during the PICU hospitalization. Patients with higher risk of mortality scores (group A) were younger than group B patients.

**Table 1:** Demographic, clinical, laboratory and markers data

**Demographic and clinical characteristics**	**Group A (n = 33)**	**Group B (n = 221)**	***P*-value**	**Overall population N = 254**
Age at admission (months)	19 (50)	45 (99)	0.012	44 (81)
Weight (kg)	11.0 (10.5)	16.0 (22.0)	0.005	15.0 (17.4)
Male sex (%)	60,6	58,8	0,853	59,1
**Admission diagnosis**	**Group A (n = 33)**	**Group B (n = 221)**	***P*-value**	**Overall population N = 254**
Postoperative	1 (1.7)	59 (98.7)	<0.001	60 (23.6)
Cardiac surgery	21 (44.7)	26 (55.3)	0.560	47 (18.5)
Respiratory	1 (2.2)	44 (97.8)	<0.001	45 (17.7)
Infectious	5 (12.5)	35 (87.5)	<0.001	40 (15.7)
Traumatic	2 (11.8)	15 (88.2)	0.002	17 (6.7)
Neurologic	10 (100)	0 (0)	-	10 (3.9)
Metabolic-Renal	1 (11.1)	8 (88.9)	0.039	9 (3.6)
Others	2 (7.7)	24 (92.3)	<0.001	26 (16.8)
PRISM III (absolute value)	13.0 (7.5)	2.0 (5.0)	<0.001	3.0 (8.0)
PIM 2 (%)	4.2 (6.7)	0.8 (1.0)	<0.001	0.9 (1.73)
**Laboratory data**	**Group A (n = 33)**	**Group B (n = 221)**	***P*-value**	**Overall population N = 254**
pH	7.26 (0.16)	7.35 (0.10)	0.001	7.34 (0.10)
Bicarbonate (mEq/L)	16.8 (8.9)	21.9 (3.9)	0.030	21.8 (3.9)
Lactate (mmol/L)	1.20 (1.13)	0.90 (1.10)	0.027	1.00 (1.10)
Platelets (X1000/mm3)	228.5 (240.4)	289.0 (169.0)	0.043	281.0 (168.0)
**Markers**	**Group A (n = 33)**	**Group B (n = 221)**	***P*-value**	**Overall population N = 254**
CRP (mg/dl)	6.23 (15.5)	1.30 (8.8)	0.210	1.47 (10.1)
PCT (ng/ml)	7.76 (25.81)	0.27 (1.36)	<0.001	0.34 (1.83)
MR-proADM (nmol/l)	1.39 (1.59)	0.53 (0.41)	<0.001	0.58 (0.39)
CT-proET-1 (pmol/l)	172.3 (82.70)	58.47 (56.50)	<0.001	67.59 (53.13)
**Markers**	**Group A1 (n = 71)**	**Group B1 (n = 182)**	***P*-value**	**Overall population N = 253**
CRP (mg/dl)	9.57 (15.4)	1.07 (7.5)	<0.001	1.47 (10.1)
PCT (ng/ml)	4.91 (32.7)	0.20 (1.28)	<0.001	0.34 (1.83)
MR-proADM (nmol/l)	1.380 (1.60)	0.471 (0.28)	<0.001	0.58 (0.39)
CT-proET-1 (pmol/l)	159.09 (97.40)	52.65 (41.00)	<0.001	67.59 (53.13)

Group A: Higher score risk mortality group. Group B: Lower score risk mortality group; Group A1: number of organ failures >1. Group A2: number of organ failures <2; admission diagnosis is described by the absolute and relative values (%); the rest of the variables are described by median and interquartile range. CRP: C-reactive protein; CT-pro-ET1: C-terminal-pro-endothelin-1; PCT:procalcitonin; MR-pro-ADM: midregional-pro-adrenomedullin.

PCT, MR-proADM and CT-proET-1 plasma levels were significantly higher in patients with higher risk of mortality scores (group A) (Table 1). CRP plasma level was not different between both groups. CRP, PCT, MR-proADM and CT-proET-1 plasma levels were significantly higher in patients with two or more number of organ failures (group A1) (Table 1).

### Value for CRP, PCT, MR-proADM and CT-proET-1 in prediction of risk of mortality scores

**Figure 1:**
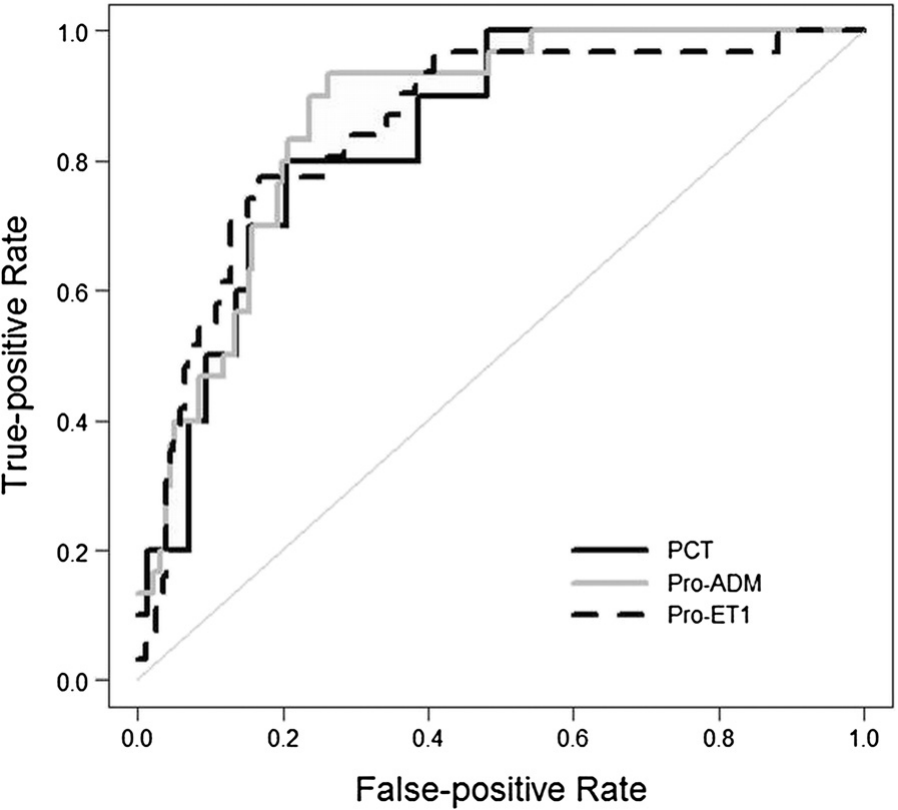
**ROC curves for PCT, MR-proADM, and CT-proET-1 in prediction of risk of mortality scores.** Legend: CRP (C-reactive protein, mg/dl), PCT (procalcitonin, ng/ml), Pro-ADM (midregional-pro-adrenomedullin, nmol/l), Pro-ET1 (C-terminal-pro-endothelin-1, units: pmol/l).

To evaluate the value for CRP, PCT, MR-proADM and CT-proET-1 in the prediction of risk of mortality scores, ROC curve analysis was performed for each pro-hormone (Table 2 and Figure 1). The optimal cut-off value of PCT for predicting risk of mortality scores was 2.05 ng/ml, showing a similar sensitivity and specificity of 80%. A MR-proADM concentration higher than 0.79 nmol/L had 93% sensitivity and 76% specificity, whereas a CT-proET-1 concentration higher than 123 pmol/L had 77% sensitivity and 84% specificity.

**Table 2:** CRP, PCT, MR-pro-ADM and CT-pro-ET-1 measurements in prediction of risk mortality scores and organ failure

**Parameters**	**AUC (95% interval confidence)**	
	**Risk mortality score**	**Organ failure (+1)**
CRP	0.608 (0.452 to 0.764)	0.713 (0.609 to 0.816)
PCT	0.839 (0.739 to 0.940)	0.804 (0.715 to 0.892)
Pro-ADM	0.866 (0.810 to 0.821)	0.922 (0.887 to 0.957)
CT-proET-1	0.853 (0.784 to 0.922)	0.915 (0.876 to 0.954)
	**Risk mortality score**	
	**Without infection**	**With infection**
CRP	0.753 (0.534 to 0.971)	0.468 (0.232 to 0.705)
PCT	0.955 (0.876 to 1.035)	0.710 (0.499 to 0.922)
MR-pro-ADM	0.886 (0.828 to 0.945)	0.869 (0.771 to 0.966)
CT-proET-1	0.893 (0.839 to 0.948)	0.856 (0.745 to 0.967)
	**Organ failure (+1)**	
	**Without infection**	**With infection**
CRP	0.770 (0.601 to 0.939)	0.634 (0.493 to 0.775)
PCT	0.868 (0.738 to 0.977)	0.739 (0.582 to 0.896)
MR-pro-ADM	0.943 (0.906 to 0.980)	0.901 (0.832 to 0.970)
CT-proET-1	0.967 (0.942 to 0.993)	0.804 (0.705 to 0.904)

Results are presented for the whole sample and for subgroups of patients with and without infection. ROC curve analysis of CRP (C-reactive protein, mg/dl), PCT (procalcitonin, ng/ml), MR-pro-ADM (midregional-pro-adrenomedullin, nmol/l) and CT-pro-ET1 (C-terminal-pro-endothelin-1, pmol/l).

### Value for CRP, PCT, MR-ProADM and CT-pro-ET1 in prediction of number of organ failures

**Figure 2:**
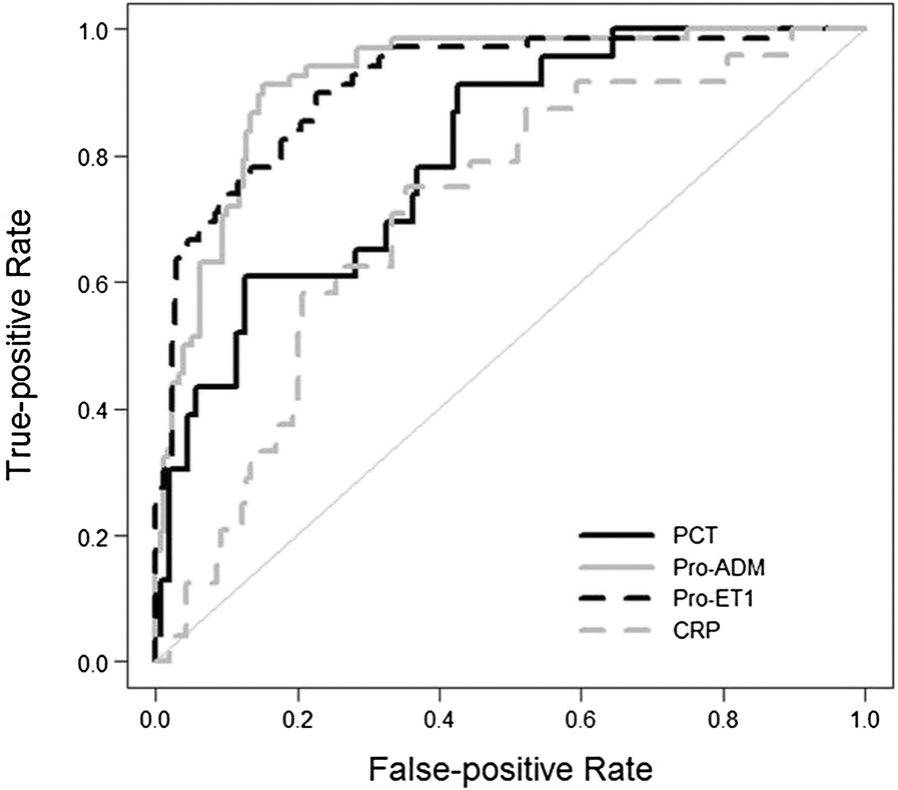
**ROC curves for CRP, PCT, MR-proADM and CT-pro-ET-1 in prediction of number of organ failures.** Legend: CRP (C-reactive protein, mg/dl), PCT (procalcitonin, ng/ml), Pro-ADM (midregional-pro-adrenomedullin, nmol/l), Pro-ET-1 (C-terminal-pro-endothelin-1, pmol/l).

ROC curve analysis was performed for each biomarker (Table 2 and Figure 2). A CRP level of 3.76 mg/dL showed a sensitivity of 75% and a specificity of 65%; a PCT level of 4.12 ng/mL gave a sensitivity of 61% and a specificity of 88%; a MR-proADM concentration higher than 0.77 nmol/L had 91% sensitivity and 85% specificity, whereas a CT-proET-1 concentration higher than 80 pmol/L had 90% sensitivity and 77% specificity.

### Subgroups analysis

Patients were classified into infectious or non-infectious subgroups during the first 24 hours after admission. We performed a subgroup analysis of mortality risk scores taking into account this classification (Table 2). In the infectious subgroup a PCT level of 2.05 ng/mL gave a sensitivity of 83% and a specificity of 58%; a MR-proADM concentration higher than 0.80 nmol/L had 100% sensitivity and 70% specificity, whereas a CT-proET-1 concentration higher than 80.57 pmol/L had 75% sensitivity and 86% specificity.

In the non-infectious subgroup a PCT level of 0.58 ng/mL gave a sensitivity of 100 and a specificity of 82, a MR-proADM concentration higher than 0.84 nmol/L had 91% sensitivity and 82% specificity, whereas a CT-proET-1 concentration higher than 83.50 pmol/L had 91% sensitivity and 70% specificity.

We also performed an infectious versus non-infectious subgroup analysis to differentiate the number of organ failures (Table 2). In the infectious subgroup a PCT level of 4.12 ng/mL gave a sensitivity of 71% and a specificity of 70%, a MR-proADM concentration higher than 0.81 nmol/L had 84% sensitivity and 79% specificity, whereas a CT-proET-1 concentration higher than 79.5 pmol/L had 79% sensitivity and 66% specificity.

In the non-infectious subgroup a PCT level of 0.40 ng/mL gave a sensitivity of 89% and a specificity of 77%, a MR-proADM concentration higher than 0.77 nmol/L had 90% sensitivity and 91% specificity, whereas a pro-ET1 concentration higher than 87.3 pmol/L had 93% sensitivity and 89% specificity.

### Analysis of quality of decision rules: cross-validation results

We performed a cross validation method to test the quality of decision rules. Results for the obtained sensitivity, specificity, PPV and NPV values are reported in Table 3.

**Table 3:** Sensitivity, specificity, positive predictive values and negative predictive values of CPR, PCT, MR-proADM and CT-pro-ET1

**Parameters**	**PICU risk mortality score**				**Organ failure (+1)**			
	**Se**	**Sp**		**PPV**	**NPV**	**Se**	**Sp**	**PPV**	**NPV**
CRP	0.416	0.640		0.072	0.942	0.583	0.642	0.192	0.914
PCT	0.700	0.798		0.167	0.979	0.610	0.569	0.169	0.910
MR-ProADM	0.900	0.758		0.337	0.982	0.897	0.850	0.693	0.956
CT-proET-1	0.710	0.741		0.386	0.954	0.884	0.768	0.592	0.946
	**PICU risk mortality score**								
	**Without infection**				**With infection**				
**Parameters**	**Se**	**Sp**		**PPV**	**NPV**	**Se**	**Sp**	**PPV**	**NPV**
CRP	0.400	0.865		0.125	0.968	0.500	0.365	0.060	0.900
PCT	0.750	0.822		0.143	0.988	0.500	0.573	0.088	0.933
MR-proADM	0.863	0.817		0.422	0.974	0.875	0.701	0.233	0.982
CT-proET-1	0.773	0.866		0.472	0.960	0.625	0.833	0.278	0.956
	**Organ failure (+1)**								
	**Without infection**				**With infection**				
**Parameters**	**Se**	**Sp**		**PPV**	**NPV**	**Se**	**Sp**	**PPV**	**NPV**
CRP	0.778	0.677		0.179	0.971	0.643	0.606	0.257	0.889
PCT	0.778	0.758		0.233	0.973	0.428	0.938	0.600	0.884
MR-proADM	0.898	0.877		0.759	0.952	0.895	0.742	0.500	0.961
CT-proET-1	0.918	0.894		0.789	0.962	0.842	0.492	0.320	0.917

Legend: Results obtained after applying a cross validation method to test the quality of decision rules, presented for the whole sample and for subgroups of patients with and without infection. CRP (C-reactive protein, mg/dl), PCT (procalcitonin, ng/ml), MR-pro-ADM (midregional-pro-adrenomedullin, nmol/l), CT-pro-ET-1 (C-terminal-pro-endothelin-1, pmol/l). Se: sensitivity. Sp: specificity. NPV: negative predictive value. PPV: positive predictive value.

### Combined score of biomarkers

We developed several scores with the best AUCs obtained with the combination of two markers. The AUC for the combination of MR-proADM and PCT in the prediction of PICU risk of mortality scores was 0.935 (0.842 to 0.978) when the decision was based on the score S1 (S1 = 1.39 · MR-proADM + 0.01 · PCT) which with a threshold of 1.70 leads to a sensitivity of 90.0% and a specificity of 90.1% (PPV and NPV were 34.6 and 99.3, respectively). The AUC for the combination of MR-proADM and CT-pro-ET1 was 0.900 (0.858 to 0.942) for decision based on the score S2 (S2 = 0.77 · MR-proADM + 0.01 · CT-pro-ET1) which with a cut-off value of 1.85 obtained a sensitivity of 90% and a specificity of 80.3% (PPV was 38.6% and NPV 98.3%). The AUC for the combination of MR-proADM and PCT in the prediction of more than 1 organ failure was 0.904 (0.848 to 0.981) when the decision was based on the score S3 (S3 = 2.04 · MR-proADM + 0.02 · PCT) which with a threshold of 2.00 achieved a sensitivity and a specificity of 82.6% and 89.2%, respectively (PPV of 52.8% and NPV of 97.2%). The AUC for the combination of MR-proADM and CT-pro-ET1 was 0.945 (0.918 to 0.973) for the score S4 (S4 = 1.53 · MR-proADM + 0.03 · CT-pro-ET1); a cut-off value of 3.65 yielded a sensitivity of 97.1% and a specificity of 79.9% (PPV and NPV were 64.7% and 98.6%, respectively). The combination of MR-proADM and CRP did not improve MR-proADM accuracy.

## Discussion

An important issue in pediatric critical care is the improvement of prognostic assessment. Markers that are able to stratify critically ill children depending on the risk of mortality could help physicians to make decisions. To our knowledge, this is the first prospective study in critically ill children which tries to identify vasoactive biomarkers as outcome predictors. We have shown that high levels of MR-proADM, CT-proET-1 and PCT are associated with increased risk of mortality scores as well as increased risk of number of organ failures in a heterogeneous sample of critically ill children. We cannot use mortality as the gold standard to differentiate a patient’s prognosis because PICU mortality was low in our sample. Therefore, we used other surrogate markers of PICU outcome, such as mortality scores. Numbers of organ failures are other criteria of severity in critically ill children. In our sample, more than 90% of the patients with a higher risk of mortality had two or more number of organ failures.

### Mortality risk

Children with higher risk of mortality scores were younger, and presented at admission with lower pH, bicarbonate and platelets, and higher lactate (Table 1). Previous studies have shown a higher risk of mortality in younger children [1,31]. Lactate levels correlated with mortality in postoperative cardiac surgery children [32,33] as well as critically ill adult patients [34]. A low level of platelets correlated with mortality increase [35].

The main objective of our study was to identify biomarkers as risk mortality predictors. CRP has been shown to be elevated in adult patients with higher mortality risk [11,12]. In agreement with other studies [9,10], we found no significant differences in CRP concentrations between group A and B patients. Slow CRP kinetics could explain this finding. PCT levels increased in direct relation to disease severity [7,36-38]. We found that a PCT cut-off value of 2.05 ng/mL predicted risk of mortality scores with 80% sensitivity and specificity. When analyzing subgroups of patients, we found that PCT was a better marker of mortality risk scores in the non-infectious subgroup (Table 2). Cut-off level decreases to 0.58 ng/mL with higher sensitivity and specificity. To explain this decrease in cut-off level and better performance we have to take into account that in patients with bacterial infection PCT increase is due to both infection and disease severity, whereas in patients without infection the only reason for a PCT increase is the acute inflammatory process from a high severity shock, trauma and so on. Therefore, when bacterial infection is absent a PCT increase could be directly related to disease severity.

To date, few data are available regarding the potential of vasoactive pro-hormones as biomarkers in mortality risk determination. MR-proADM was evaluated as a severity marker in septic adult patients with promising results [39]. Increased admission levels of MR-proADM in critically ill patients have been associated with increased mortality [39,40]. We found that MR-proADM can be useful to identify critically ill children with different risk of mortality scores. Areas under the ROC curve range from 0.67 in the study of Schuetz *et al*. [15] to 0.87 in the Wang *et al*. study [40] with an intermediate value of 0.81 in the study of Christ-Crain *et al*. [18] in patients with sepsis and septic shock. We obtained a value of 0.86. Our MR-proADM cut-off value of 0.79 nmol/L was similar to values of 0.95 described to predict mortality in community-acquired pneumonia patients [41] but lower than the value of 3.9 nmol/L obtained by Christ-Crain *et al*. [18] in septic patients. The reason could be different patients and different methodology. In our patients, we tried to differentiate risk of mortality scores at the level of p75 whereas Christ-Crain *et al*. [18] tried to differentiate survival versus nonsurvival patients. We only found a pediatric study in septic neonates comparing MR-proADM levels between survivors and nonsurvivors. MR-proADM levels were 5.22 nmol/L and 11.40 nmol/L, respectively [42]. However, these values had to be analyzed with caution due to other factors that can increase MR-proADM in neonatal patients.

CT-proET-1 was also a good marker of mortality risk with AUCs under the ROC curve similar to MR-proADM. Brauner *et al*. [21] also found an association between CT-proET-1 levels and mortality in septic shock patients. Other adult studies did not find differences in CT-proET-1 levels between survivors and non-survivors [15,43].

Different from PCT, MR-proADM and CT-proET-1 areas under ROC curve were similar in patients with and without infection. Moreover, PCT cut-off levels were different in infectious versus non-infectious patients, whereas MR-proADM and CT-proET-1 cut-off levels were almost the same in both groups of patients. This is an advantage for both vasoactive peptides that were not influenced by the infectious situation of the patient.

### Number of organ failures

The performance of biomarkers to identify patients with two or more organ failures was similar to that previously described for risk of mortality, except for CRP. We found higher serum CRP levels in children from group A1 versus B1. Lobo *et al*. [11] showed a higher incidence of respiratory, renal and hematologic organ failures in patients with CRP levels higher than 10 ng/mL. Our cut-off value to differentiate groups A1 and B1 was 3.76 mg/dl. Values for the areas under the ROC curve to differentiate group A1 versus B1 were acceptable for CRP, good for PCT and excellent for CT-proET-1 and MR-proADM (Table 2 and Figure 2). PCT cut-off values of 4.12 ng/mL were higher than previous values for mortality risk whereas MR-proADM cut-off values of 0.77 nmol/L were similar and CT-proET-1 cut-off values of 80.57 pmol/L were lower. Several studies found a correlation between PCT increase during the first 24 hours after admission [44] or postoperative PCT increase [45] and number of organ failures. Ueda *et al*. [46] showed a correlation between MR-proADM levels and the multiple organ failure score.

### Analysis of quality of decision rules: cross-validation results

As expected, the values of sensitivity, specificity, NPV and especially PPV were lower after performing a cross-validation test. However, the information given by the markers, especially MR-proADM, appears to improve diagnostic accuracy for detection of patients with higher risk of mortality scores and more than one organ failure.

### Combined score of biomarkers

The single marker with the greatest AUC was MR-proADM. Combination of MR-proADM with each of the other markers slightly improves diagnostic efficiency to differentiate groups. We have developed several scores with the best AUCs obtained with the combinations of MR-proADM and PCT, and MR-proADM and CT-proET1.

### Prognostic assessment

The additional value of information provided by biomarkers when compared with scoring systems has to be further evaluated in order to translate their measurements into clinical practice. PRISM III and PIM 2 scores have been validated for mortality risk stratification, but tend to be used more for audit and research than clinical decision making [1,3-5]. A rapidly available biochemical test that provides similar prognostic information could, therefore, be useful, for example, to help discussions about prognosis with patients’ relatives and decisions regarding earlier interventions. Future work should clarify whether the increase of MR-proADM and CT-proET-1 in pediatric patients with higher risk of mortality results from an up-regulation of biomarker secretion in patients with different organ failures. A rising PCT level is currently used as an indicator that an infectious process is not under control and that better source control is required [47]. Future work should clarify whether PCT at a low cut-off level could be used for prognosis in non-infectious patients.

Our study has limitations. First, taking into account that it was not sufficiently powered to pick up differences in survival, we used other surrogate markers of PICU outcome, such as mortality scores, against which the biomarkers diagnostic criteria can be calibrated. Second, we have performed an observational study that does not allow drawing any conclusion concerning therapeutic interventions. Third, this was a two-center study that includes different groups of critically ill children. The value for biomarkers in prediction of adverse outcome would be different if the population was different. Fourth, biomarker levels were analyzed during the first 12 hours after PICU admission, but follow-up measurements were not available. Evolution of biomarker levels during the first admission days would have higher accuracy. However, an early mortality prediction is more useful in clinical practice. Fifth, the A and B groups were different in terms of diagnosis. We cannot totally rule out that this difference has influenced biomarkers levels.

## Conclusions

High levels of MR-proADM, CT-proET-1 and PCT were associated with increased prediction of mortality risk scores. MR-proADM, CT-proET-1 and PCT concentrations higher than 0.80 nmol/L, 123 pmol/L and 2 ng/mL, respectively, could be used by clinicians to identify critically ill children at higher prediction of death risk score.

## Key messages

• MR-proADM, CT-proET-1 and PCT levels during the first 12 hours after PICU admission are associated with increased prediction of mortality risk score in critically ill children.

• The cut-off level to identify critically ill children at higher score risk of death were 0.80 nmol/L for MR-proADM, 123 pmol/L for CT-proET-1 and 2 ng/mL for PCT.

• The additional value of these combinations of biomarker cut-off levels when compared with scoring systems has to be further evaluated in order to translate their measurement into clinical practice.

## Abbreviations

ADM: adrenomedullin; AUC: area under curve; CRP: C-reactive protein; CT-pro-ET-1: C-terminal-pro-endothelin-1; ET-1: endothelin-1; MR-proADM: midregional-pro-adrenomedullin; p75: 75 percentile; PCT: procalcitonin; PICU: pediatric intensive care unit; PIM 2: Pediatric Index of Mortality 2; PRISM III: Pediatric Risk of Mortality III; ROC: Receiver Operating Characteristic; SIRS: systemic inflammatory response syndrome.

## Competing interests

Corsino Rey has received speaker honoraria from Brahms and Thermofisher Companies to attend meetings related to sepsis biomarkers. The other authors declare that they have no conflicts of interest.

## Authors’ contributions

CR participated in protocol conception and design, obtaining funding, subject enrollment (PICU patients), data collection, interpretation of data and wrote the first draft of the manuscript. IG-H participated in subject enrollment, data collection and interpretation of data. AC participated in subject enrollment, data collection and drafting the manuscript. PM-C participated in study design, statistical data analysis and interpretation. MB participated in subject enrollment of Hospital Universitario Gregorio Marañón patients, data collection and drafting the manuscript. AM participated in subject enrollment, data collection and drafting the manuscript. BP participated in laboratory sample management and interpretation of data. JL-H participated in protocol conception and design, interpretation of data and drafting the manuscript. All authors read and approved the final manuscript.
